# Report of a Case of Diphtheritic Paralysis

**Published:** 1864-08

**Authors:** J. G. Meachem

**Affiliations:** Racine, Wis.


ART. II.—*Report of a case of Diphtheritic Paralysis*, BY J. G. MEACHEM, M. D., *Racine, Wis.*

Melissa C——, aged 18 years, called at my office for advice on the 19th of September. She presented the following symptoms: severe pain in the head, back and limbs, alternation of chills and fever, sore throat, and painful diglution. The tonsils, soft palate, and posterior fauces were covered with a heavy buckskin colored diphtheric exudation; the breath fetid; pulse rapid, and a degree of general prostration present, unusual for the length of time that she had been ill. She had walked a distance of three blocks, but was entirely overcome by the exertion. The first symptoms of indisposition manifested themselves the day before. I at once applied, by means of a camel's hair brush, a solution of the ferri per sulphatis to every part covered with the exudation. She, was directed to take a teaspoonful of the following mixture every four hours:

| | | |
|---|---|---|
| ℞ Chl. Potass, | ʒ i. | |
| Aqua Pura, | ℥ iv. | |
| Tr. Ferri Mur, | ʒ i. | M. |

Two grains of quinine was also given between each dose of the mixture, and a soft flannel, saturated with turpentine, applied to the external throat.

Sept. 20th. Symptoms same as yesterday, with the exception that she has no chills, but has a continued fever. The solution of iron was again applied, and the other treatment continued.

21st. Is decidedly better. Exudation almost entirely disappeared; no fever; no fetor of breath. Treatment continued.

22d. Is to all appearance well. Patient discharged.

On the afternoon of the 26th I was again called to visit my patient. That morning she had imprudently assisted in doing a washing, and still more rashly, after finishing, when the system was fatigued and relaxed from the exertion, walked half a mile facing a strong, cold, north wind, with no protection whatever to the throat, having that morning cast aside a thick covering of cotton batting which had been worn since dispensing with the turpentine some days before. This imprudence resulted in bringing upon her another severe chill, which lasted nearly two hours. It was followed by violent reaction, and inflammation of the tonsil glands, palate and tongue. The swelling was so great as totally to prevent deglutition. After four days' contiuuance an abscess formed in each tonsil, both of which were opened through the palate, giving vent to a large quantity of very fetid pus; she was greatly relieved, and in a few hours after drank freely of beef tea.

Oct. 1st. The swelling has somewhat subsided, but the throat is again covered with diphtheritic exudation, which is the first seen since the former attack. She was now put upon chl. potass and hydrochloric acid mixture and quinine, and the throat washed as before with the solution ferri per sulphatis. She has swallowed nothing to-day as nutriment.

Oct. 2d. Exudation a little less; prostration considerable; pulse rapid and feeble. Continued the solution of iron to the throat, but omit the mixture and quinia, as the patient is utterly unable to swallow anything. She has made many efforts to get down a little water, but each time it is expelled at the nostrils. She was now ordered an enema of three ounces of essence of beef every six hours, and two grains of quinine were to be added to each injection.

Oct. 3d. The enemas have been retained, and from their effect she feels a little stronger. She now has aphonia almost complete. She has renewed her efforts at deglutition, but without success. From this time forward until the 9th of November, when she expired, she was sustained entirely by the beef injections. The quantity was largely increased, so that upon the average, three pounds daily were used in making the essence. The enemas were well retained until two o r three days prior to dissolution, when occasionally one would pass off almost immediately after being thrown up. Twice during her sickness I passed the stomach tube into the stomach, and injected a pint of milk and water, but the effort taxed her feeble powers so severely that I was obliged to desist from its further use. To overcome the paralysis I resorted to stimulating frictions to the throat, blisters to the upper spine, strychnia per rectum and endermically, electricity, etc., but all to no effect, for she has swallowed nothing since Oct. 3d.

Two points in this case are worthy of note. First, that the paralysis of the throat consequent upon diphtheritic poison caused it to result fatally, and second that the was sustained so long by the injections, and retained such quantities. The essence of 114 pounds of beef having been used during the thirty-eight days; truly a large quantity to be absorbed by the rectal mucous membrane.

The chief sufferings of this patient was from intense thirst. The day before her death she drew into her mouth, through a glass tube, two pailsful of cold water. As soon as the mouth was full she would allow it to run out, and after she became so feeble that she could not suck the water from the tube, she would protrude her tongue, and ask the attendants to pour the fluid upon it from a pitcher, and would not be satisfied unless there was a stream constantly falling upon it.

